# Gold Nanoparticles Inhibit Steroid-Insensitive Asthma in Mice Preserving Histone Deacetylase 2 and NRF2 Pathways

**DOI:** 10.3390/antiox11091659

**Published:** 2022-08-26

**Authors:** Magda F. Serra, Amanda C. Cotias, Andreza S. Pimentel, Ana Carolina S. de Arantes, Ana Lucia A. Pires, Manuella Lanzetti, Jandir M. Hickmann, Emiliano Barreto, Vinicius F. Carvalho, Patrícia M. R. e Silva, Renato S. B. Cordeiro, Marco Aurélio Martins

**Affiliations:** 1 Laboratory of Inflammation, Oswaldo Cruz Institute, Oswaldo Cruz Foundation, Rio de Janeiro 21040-360, RJ, Brazil; 2 Institute of Biomedical Science, Federal University of Rio de Janeiro, Rio de Janeiro 21941-902, RJ, Brazil; 3 Institute of Physics, Federal University of Rio Grande do Sul, Porto Alegre 91509-900, RS, Brazil; 4 Laboratory of Cell Biology, Federal University of Alagoas, Maceió 50072-900, AL, Brazil

**Keywords:** gold nanoparticle, steroid-insensitive asthma, lung inflammation, NRF2, HDAC2, oxidative imbalance

## Abstract

Background: Gold nanoparticles (AuNPs) can inhibit pivotal pathological changes in experimental asthma, but their effect on steroid-insensitive asthma is unclear. The current study assessed the effectiveness of nebulized AuNPs in a murine model of glucocorticoid (GC)-resistant asthma. Methods: A/J mice were sensitized and subjected to intranasal instillations of ovalbumin (OVA) once a week for nine weeks. Two weeks after starting allergen stimulations, mice were subjected to Budesonide or AuNP nebulization 1 h before stimuli. Analyses were carried out 24 h after the last provocation. Results: We found that mice challenged with OVA had airway hyperreactivity, eosinophil, and neutrophil infiltrates in the lung, concomitantly with peribronchiolar fibrosis, mucus production, and pro-inflammatory cytokine generation compared to sham-challenged mice. These changes were inhibited in mice treated with AuNPs, but not Budesonide. In the GC-resistant asthmatic mice, oxidative stress was established, marked by a reduction in nuclear factor erythroid 2-related factor 2 (NRF2) levels and catalase activity, accompanied by elevated values of thiobarbituric acid reactive substances (TBARS), phosphoinositide 3-kinases δ (PI3Kδ) expression, as well as a reduction in the nuclear expression of histone deacetylase 2 (HDAC2) in the lung tissue, all of which sensitive to AuNPs but not Budesonide treatment. Conclusion: These findings suggest that AuNPs can improve GC-insensitive asthma by preserving HDAC2 and NRF2.

## 1. Introduction

Asthma is a non-communicable chronic pulmonary disease recognized as a significant worldwide health concern [1]. It is a highly complex and heterogeneous dysfunction associated with airway inflammation, variable airflow limitation, mucus exacerbation, airway hyper-reactivity, and sometimes lung remodeling [2]. Epidemiologic evidence shows that the disease affects individuals of all ages, with a global prevalence of over 300 million people and can be fatal, causing 250,000 annual deaths [1].

Current guidelines recommend that asthmatics be regularly treated with low doses of inhaled steroidal anti-inflammatory drugs, the most effective asthma therapy available so far [1,3]. Still, about 10% of patients require the maximal inhaled corticosteroid dose or elevated doses of oral corticosteroids, which leads to substantial adverse effects. Moreover, approximately 1% of asthma patients is entirely corticosteroid insensitive, increasing vulnerability to intensified chronic inflammatory response [2,3], and prompting the search for novel anti-inflammatory treatments [2].

The clinical application of gold metallic compounds and their complexes for treating inflammation-related diseases has several thousand years of history [4,5], but some side adverse effects limit its use [6]. The innovative nanomedicine concept, which refers to biologically active molecules formulated as nanoparticles for improving bioavailability, pharmacokinetics, biodistribution, and metabolization of drugs, has attracted growing interest. Nanomaterials are widely recognized as a way of concentrating active molecules in selected tissue targets minimizing side effects [7,8]. Notably, gold nanoparticles (AuNPs) possess significant anti-inflammatory and anti-oxidative properties and have been associated with therapeutic effects on several chronic pathological conditions, including cancer [9,10], sepsis [11], and rheumatoid arthritis [12]. The mode of action is not entirely understood, but activation of NRF2-dependent pathways [13] and the AuNPs’ potential in mimicking the activity of antioxidant enzymes, such as catalase and superoxide dismutase [14], might play a pivotal role in this protective effect.

Previous studies by our group demonstrated that the intranasal instillation of 13 nm citrate conjugate AuNPs prevents pivotal pathological changes in corticosteroid-sensitive short-term murine models of asthma in a mechanism associated with down-regulation of oxidative stress and cytokine/chemokine production [15]. Since oxidative imbalance and pro-inflammatory cytokines have an essential role in causing corticosteroid insensitiveness [3,16,17], we hypothesized that AuNPs would present a protective effect on corticosteroid-resistant asthma.

In this study, we describe a murine model of asthma in which repeated allergen exposure of sensitized A/J mice for nine consecutive weeks causes steroid-insensitive asthma changes. All these changes are sensitive to AuNPs given by nebulization, in a mechanism associated with down-regulation of oxidative stress and rescue of histone deacetylase 2 (HDAC2) levels.

## 2. Materials and Methods

### 2.1. Synthesis and Characterization of AuNPs

AuNPs were prepared and characterized as previously reported [15]. Briefly, a 0.5 mL aliquot of 25 mM HAuCl4 · 3H_2_O was added into a 250 mL round-bottom flask containing with 49 mL of Milli-Q water. The solution was heated, stirring until boiling, and then 1.5 mL of 1% aqueous sodium citrate was added. The boiling and stirring were continued for 30 min, at which point a wine-red solution was obtained. After cooling to room temperature, the AuNP solution was diluted to 50 mL using deionized water to replace the water evaporated during boiling. We used small angle X-ray scattering (SAXS) and zeta-potential measurements (Malvern Zetasizer Nano ZS system) to determine the shape, size, dispersity, and average zeta-potential of citrate-stabilized AuNPs used in this study. SAXS experiments were carried out at the D1B-SAXS1 beamline at the LNLS for 0.05 mg/mL and the characteristic SAXS intensity profile was typical of scattering objects consisting of widely separated homogenous hard spheres. The average radius of the particles was 6.3 nm with a standard deviation of 0.10 nm. The high quality of the fittings in the low-q range of the SAXS profile indicates the absence of particle aggregates. The average zeta-potential was −35.0 ± 2.0.

### 2.2. Animals and Experimental Procedures

Male A/J mice aged 6–8 weeks were obtained from the Oswaldo Cruz Foundation Breeding Center and provided water and food *ad libitum* throughout the experiment. Mice were treated according to the protocols approved by the Committee on Use of Laboratory Animals of the Oswaldo Cruz Foundation (Rio de Janeiro, Brazil; license number, CEUA L-002/2020-A2). The animals were equally and randomly distributed into five groups: (i) sensitized and challenged with NaCl sterile solution (saline), (ii) sensitized and challenged with ovalbumin (OVA) and treated with saline, (iii) sensitized and challenged with OVA and treated with Budesonide, iv) sensitized and challenged with OVA and treated with gold nanoparticle (13 nm AuNPs–0.4 μg/mL) stabilized by citrate species prepared as previously reported [15] and (v) sensitized and challenged with ovalbumin OVA and treated with gold nanoparticle (13 nm AuNPs–4 μg/mL).

To generate the chronic asthma model of steroid resistance, mice were sensitized by a dorsal subcutaneous injection of OVA (50 μg) in 5 mg of aluminium hydroxide on day 0 and day 7. On day 14 post-sensitization, animals were intranasally challenged with OVA (50 µg/25 µL saline) for nine consecutive weeks, once a week. Three weeks after the beginning of OVA challenges, mice were placed in a Plexiglas chamber (45 × 28 × 29 cm) and subjected to daily interventional nebulization, 1 h before the challenge of either 0.4 or 4 μg/mL AuNPs or Budesonide solution (7.5 mg/mL; compound in 0.5% methanol: 0.5% Tween 80: 99% 0.9% NaCl) as reported [18] or an equal volume of vehicle for 30 min using an air-driven nebulizer (model NS I -210/12; Indústria de Aparelhos Biomédicos, Brazil) at a flow rate of 2.5 L/min.

### 2.3. Invasive Assessment of Respiratory Mechanics

Airway responsiveness was determined as a change in airway function 24 h after the last challenge with aerosolized methacholine in a FinePointe R/C Buxco Platform (DSI Harvard Bioscience, Inc.—St. Paul, MN, USA). Mice were anesthetized, tracheostomized, and mechanically ventilated, and transpulmonary resistance and elastance were assessed as previously described [18]. Analogical signals from the computer were digitized using a Buxco Analog/Digital Converter. Mice were allowed to stabilize for 5 min, and increasing concentrations of methacholine (3, 9 and 27 mg/mL) were aerosolized for 5 min each. Baseline pulmonary parameters were assessed with aerosolized phosphate-buffered saline (PBS). Expressed results comprised the mean absolute values of the responses of lung resistance (cm H_2_O/mL/s) and elastance (mL/cm H_2_O) collected 5 min after the administration of methacholine aerosol.

Only for this assay, we used mice intranasally challenged with OVA for 4 or 9 consecutive weeks, once a week. The Budesonide treatment protocol was the same as described above.

### 2.4. Lung Tissue Digestion and Leucocyte Enumeration

The A/J sensitized mice lungs perfused with saline containing 20 mM EDTA were immediately excised, weighed, cut into pieces, and digested with 1 mL of 0.2% collagenase diluted in RPMI 1640 for 40 min at 37 °C to obtain lung leucocytes. The mixture was then filtrated through a 200-mesh stainless-steel gauze supplemented with RPMI and centrifuged at 300× *g* for 10 min. The cell pellet was resuspended in 1 mL RPMI media. Total leucocyte enumerations were carried out in Neubauer chambers using light microscopy after dilution of the samples of lung cells in Türk’s (acetic acid, 0.2%) solution. Mononuclear cells, eosinophils and neutrophils were counted using standard morphologic criteria from cytocentrifuge preparations stained with May–Grünwald–Giemsa stain and expressed as cell/mg lung.

### 2.5. Determination of Myeloperoxidase Activity in the Lung (MPO)

MPO activity, a marker of neutrophil infiltration, was determined using one-third of the right lung lobe. Cryopreserved lung tissue samples were homogenized (Homogenizer; Omni International, Kennesaw, GA, USA), and the corresponding extracts were centrifuged at 10,000× *g* for 10 min. The pellets were suspended in PBS supplemented with HTAB (0.5%) and EDTA (5 mM), and the resulting supernatants were isolated after centrifugation. The supernatants were plated with PBS-HTAB-EDTA, HBSS, O-dianisidine dihydrochloride (1.25 mg/mL), and 0.4 mM H_2_O_2_. After 15 min of incubation at 37 °C in an agitator, the reaction was stopped by adding NaN3 (1%). The MPO activity was measured and normalized to protein content and expressed as an optical density of 460 nm.

### 2.6. Histopathological Evaluation of Pulmonary Inflammation and Airway Remodeling

The left lung lobes were removed and fixed in Millonig buffer solution (pH 7.4) with 4% paraformaldehyde. Histologic 4 μm-thick sections were stained with Llewellyn’s Sirus Red (Direct Red 80, CI 35780; Aldrich, Milwaukee, WI, USA) for quantitative analyses of peribronchiolar eosinophils and neutrophils. Cell analyses were made in six randomly selected fields at a magnification of X 1000, counted according to their morphological criteria, and expressed as cells/area (10^4^/µm^2^) [19]. Additionally, periodic acid-Schiff (PAS-Sigma-Aldrich) (St. Louis, MO, USA) revealed mucus production. The lung section revealed the total mucus area between the airway epithelial basal membrane and the inner epithelium layer of 10 distal airways. Results were expressed as PAS-positive area (pixel/µm^2^). The extension of peribronchiolar Gömöri’s trichrome staining (Sigma-Aldrich) was measured between the airway epithelial basal membrane and airway adventitia and quantified for the total deposition of the extracellular matrix. The slides were coded, and the analyses were carried out blindly. Random airways were photographed using a light microscope Olympus BX40 (Olympus, PA, USA) at x 400 and a digital camera [19]. Results were expressed as extracellular matrix deposition area (pixel/µm^2^). The evaluations were performed using standard image analysis protocol using an image analyzer system (Image-Pro**^®^** Plus, 4.1; Media Cybernetics, Houston, TX, USA).

### 2.7. Cytokine and Chemokine Measurements

Cytokines (interleukin (IL)-4, IL-13, and IL-17) and chemokines (CCL11/eotaxin-1, CCL24/eotaxin-2, CXCL1/keratinocyte chemoattractant (KC) and CCL17/thymus and activation regulated chemokine (TARC)) were measured in lung homogenates using commercially available enzyme-linked immunosorbent assay kits according to the manufacturer׳s instructions. Lung tissue was homogenized on ice using a Tissue Tearor (Omini mH homogenizer) in PBS containing Triton X-100 and a protease inhibitor mixture, as previously described [19]. The absorbance of each sample was measured at 450 nm using a Bio-Rad Model 680 microplate reader. Cytokine and chemokine protein levels were determined from standard curves and are expressed in pg/mg of tissue.

### 2.8. Redox Parameters

As described above, the catalase and lipid peroxidation activities were measured in lung homogenates prepared samples. For these assays, the total protein in the lung homogenates was determined by the Bradford method using bovine serum albumin as the standard. Catalase activity was measured by quantifying the rate of decay of hydrogen peroxide at 240 nm, and it was expressed as U/mg protein [20]. For the evaluation of the lipid peroxidation marker, the thiobarbituric acid reactive substances (TBARS) method was performed to analyze malondialdehyde (MDA) levels [21]. TBARS were determined by absorbance at 532 nm and expressed as MDA nmol/mg of protein.

### 2.9. Western Blotting

Nuclear protein fractions were isolated from lung tissues using TransAM^®^ according to the manufacturer’s instructions (Active Motif, Rixensart, Belgium) to detect the expression of HDAC2. The lung whole-cell extracts were evaluated to see the expression of NRF2 and PI3Kδ using Western blot analysis. Samples (50 μg protein concentration) were separated by SDS-PAGE and electroblotted onto nitrocellulose membranes. Primary antibodies employed were anti-HDAC2 (H2663/Sigma-Aldrich), Lamina-B (sc-6217/ Santa Cruz Biotechnology) (Dallas, TX, USA), anti-NRF2 (SAB2501713/Sigma-Aldrich) (St. Louis, MO, USA), a β-actin (AB8226/ABCAM) (Cambridge, UK) anti-PI3Kδ (EPR386/ABCAM) (Cambridge, UK) and GAPDH (SC20358/Santa Cruz Biotechnology) (Dallas, TX, USA). The membranes were incubated with HRP-conjugated anti-mouse IgG secondary Ab (no. HAF007; R&D Systems) (Mineápolis, MN, USA) or HRP-conjugated anti-rabbit IgG secondary Ab (no. HAF008; R&D Systems) (Mineápolis, MN, USA) for 1 h at room temperature. Electrochemiluminescence and a Western blotting detection system with subsequent exposure to X-ray film (Kodak, Rochester, NY, USA) visualize the membrane. Western blotting images were analyzed by densitometry using Image J (National Institutes of Health, software version 1.53K, Bethesda, MD, USA) [22].

### 2.10. Statistical Analyses

Data were analyzed using GraphPad Prism (GraphPad Software, version 9.4.0, San Diego, USA), and results were expressed as the mean ± the standard error of the mean (SEM). To determine the significance pf data with multiple comparations, we used one-way ANOVA was used followed by the Newman–Keuls Student test. The *p* values of 0.05 or less were considered significant.

## 3. Results

### 3.1. Effect of AuNPs but Not Budesonide on Airway Hyper-Reactivity (AHR) Caused by Allergen Provocation

**Figure 1:**
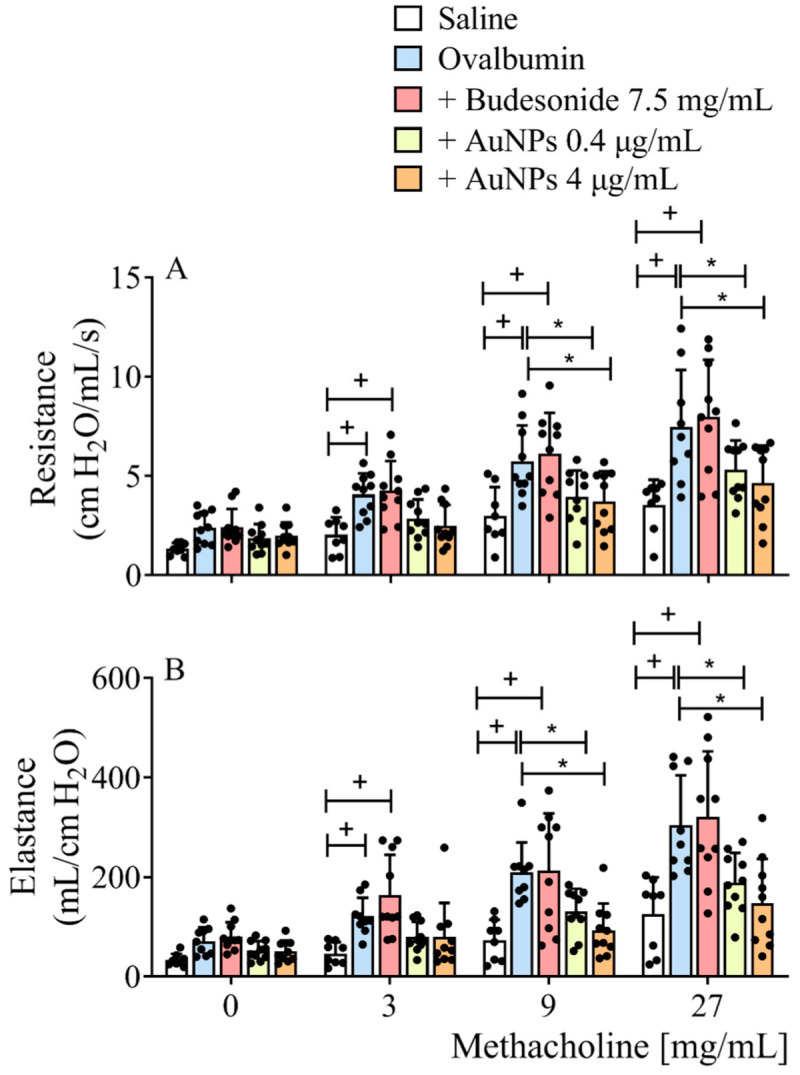
The effect of treatment with nebulized Budesonide or gold nanoparticles (AuNPs) on airway hyper-reactivity following the protocol of ovalbumin intranasal instillation given once a week for 9 weeks. The treatment was given 1 h before allergen provocation at weeks 3 to 9. Airway responsiveness was measured by changes in lung resistance and elastance induced by aerosolization of increasing concentrations of methacholine 24 h after the last antigen challenge. Data are expressed as mean ± SD (Each dot represents an individual mouse; *n* of at least 8 mice per group). + *p* ˂ 0.05 as compared with the saline group, * *p* ˂ 0.05 as compared with the ovalbumin-challenged group.

Figure 1 shows that sensitized A/J mice exposed to intranasal instillations of allergen (OVA) for 9 weeks responded with substantial airway hyper-reactivity, as revealed by elevated values of airway resistance (Figure 1A) and lung elastance (Figure 1B) following exposure to methacholine when compared with sham-challenged mice (black vs. white columns). Figure 1 also shows that nebulized Budesonide (7.5 mg/mL, 30 min) failed to inhibit AHR under conditions where AuNPs (0.4 e 4 μg/mL, 30 min) were active. It is noteworthy that despite being unable to alter AHR caused to allergen exposure in this long-term asthma model, Budesonide did inhibit this response in mice subjected to a shorter-term version of it (only 4 instead of 9 weeks of allergen provocations) (Figure S1 in the online Supplementary Material).

### 3.2. Effect of AuNPs but Not Budesonide on Inflammatory Changes Caused by Allergen Provocation

**Figure 2:**
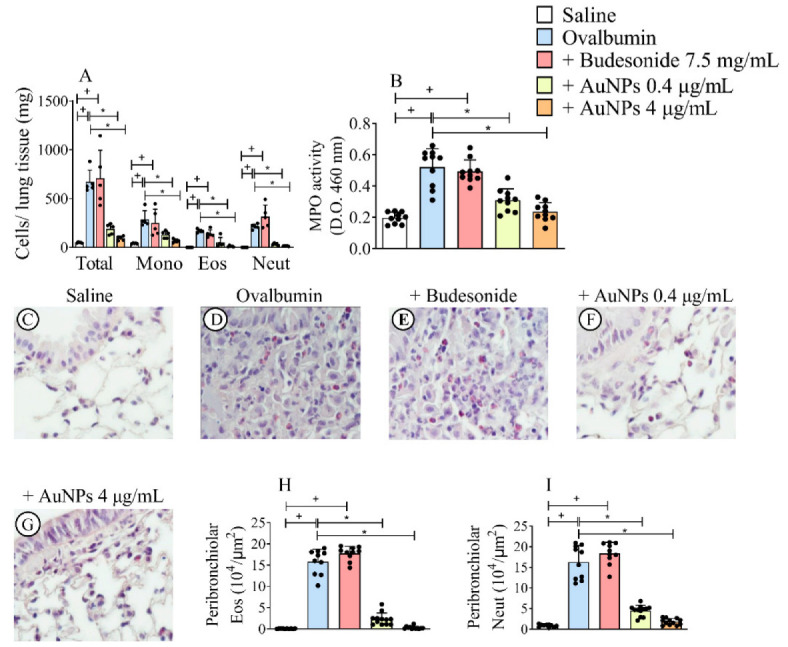
Effect of nebulized Budesonide or gold nanoparticles (AuNPs) on lung inflammation following the protocol of ovalbumin intranasal instillation given once a week for 9 weeks. Data shown represent changes in inflammatory cell numbers in lung tissue digests (**A**) and lung tissue myeloperoxidase (MPO) activity (**B**) 24 h after the last allergen challenge. Photomicrographs of paraffin-embedded Sirius Red–stained lung sections from ovalbumin-sensitized mice challenged with saline (**C**), challenged with ovalbumin (**D**), challenged with ovalbumin, and treated with Budesonide (**E**), challenged with ovalbumin and treated with either AuNPs (0.4 μg/mL) (**F**) or AuNPs (4 μg/mL) (**G**), respectively. The number of eosinophils (Eos) (**H**) and neutrophils (Neut) (**I**) in peribronchiolar regions was determined in Sirius Red-stained lung sections (**C**–**G**) by morphometric analyses. Data are expressed as mean ± SD (Each dot represents an individual mouse; *n* = 10 for all analyses except the one concerning changes in inflammatory cell numbers in lung tissue digests which has *n* = 5). + *p* ˂ 0.05 as compared with the saline group, * *p* ˂ 0.05 as compared with the ovalbumin-challenged group.

The analyses of the inflammatory cellular infiltrate were performed by (i) enumeration of total and differential leukocyte counts in lung tissue digested samples, (ii) MPO activity of lung tissue samples, and (iii) peribronchiolar histologic examinations undertaken 24 h after the last allergen provocation. As shown in Figure 2A, compared with sham-challenged control animals, allergen stimulation caused a marked increase in the total leukocyte numbers in lung digested samples, which remained unaltered in mice treated with Budesonide, but appeared reduced in mice subjected to AuNPs.

The increase in total leukocyte numbers was accounted for by the rise in the levels of mononuclear cells, eosinophils, and neutrophils, all of which were inhibited by treatment with AuNPs, but not Budesonide (Figure 2A). Similar findings were achieved following the colorimetric MPO analysis as a surrogate for neutrophil numbers. Allergen-induced elevation in MPO activity appeared sensitive to AuNPs, at both doses employed, under Budesonide refractoriness conditions (Figure 2B). Histologic evaluation of the lungs of saline-challenged mice (Figure 2C) and ovalbumin-challenged mice (Figure 2D) revealed intense peribronchiolar inflammatory infiltration after allergen provocation that was not sensitive to Budesonide (Figure 2E). Still, it was sensitive to AuNPs 0.4 μg/mL (Figure 2F) and 4 μg/mL (Figure 2G). Quantitative morphometric analyses of lung sections showed that nebulized AuNPs, but not Budesonide, significantly inhibited the infiltration of eosinophils (Figure 2H) and neutrophils (Figure 2I).

### 3.3. AuNPs Inhibit Allergen-Induced Mucus Production and Peribronchiolar Collagen Deposition Caused by Allergen Provocation

**Figure 3:**
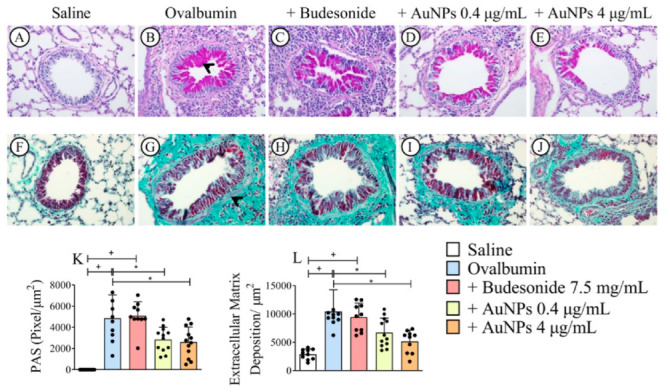
The effect of nebulized Budesonide or gold nanoparticles (AuNPs) on mucus production and airway remodeling following ovalbumin intranasal instillation given once a week for 9 weeks. Photomicrographs were taken of representative airways stained by periodic acid-Schiff (**A**–**E**) or Gömöri trichrome (**F**–**J**) from ovalbumin-sensitized mice challenged with saline (**A**,**F**), challenged with ovalbumin (**B**,**G**), challenged with ovalbumin and treated with Budesonide (**C**,**H**), challenged with ovalbumin and treated with AuNPs (0.4 μg/mL) (**D**,**I**) and challenged with ovalbumin and treated with AuNPs (4 μg/mL) (**E**,**J**), respectively. Quantitative assessments of mucus production (**K**) and fibrotic changes (**L**) were carried out in lung sections by morphometric analyses. Data are expressed as mean ± SD (Each dot represents an individual mouse; *n* = 10 for all groups). + *p* ˂ 0.05 as compared with the saline group, * *p* ˂ 0.05 as compared with the ovalbumin-challenged group.

**Figure 4:**
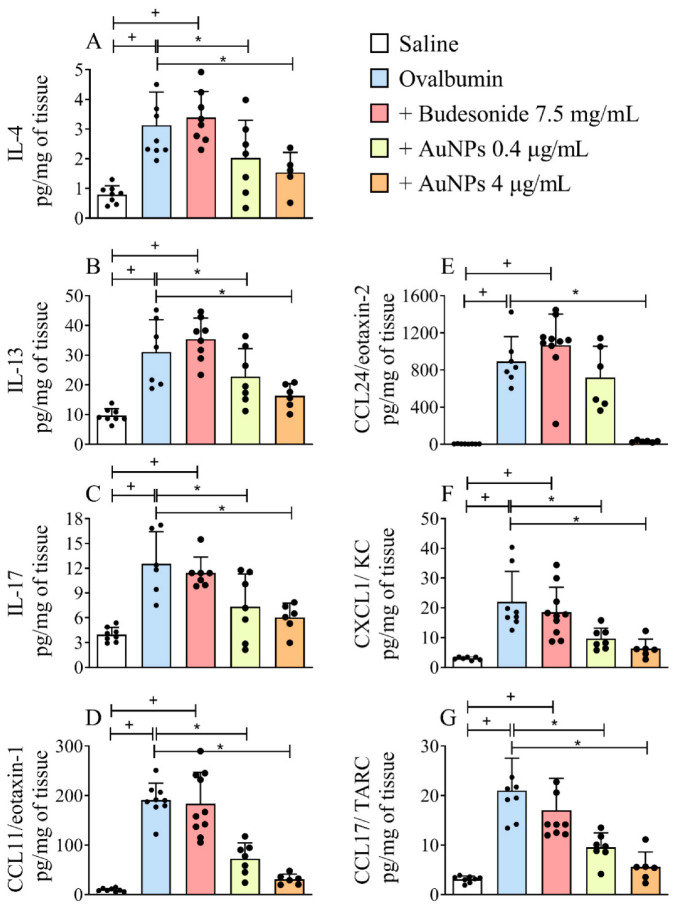
The effect of nebulized Budesonide or gold nanoparticles (AuNPs) on changes in cytokine and chemokine levels following ovalbumin intranasal instillation given once a week for 9 weeks. The levels of interleukin (IL)-4 (**A**), IL-13 (**B**), IL-17 (**C**), CCL11/eotaxin-1 (**D**), CCL24/eotaxin-2 (**E**), CXCL1/ keratinocyte chemoattractant (KC) (**F**) and CCL17/ thymus and activation regulated chemokine (TARC) (**G**) were measured by Enzyme-Linked Immunosorbent Assay (ELISA) in whole-lung homogenates. Data are expressed as mean ± SD (Each dot represents an individual mouse; *n* = 6 for all analyses, except IL-4 quantification under AuNP treatment, whose *n* is 5). + *p* ˂ 0.05 as compared with the saline group, * *p* ˂ 0.05 as compared with the ovalbumin-challenged group.

Lung sections stained with the periodic acid-Schiff stain revealed an extensive mucus production following allergen stimulation. The representative photomicrograph of sham-challenged mice (Figure 3A) shows no significant mucus production, whereas that from allergen-challenged mice (Figure 3B) exhibited mucus hypersecretion (arrowhead) within the airway epithelia. Budesonide failed to alter the response to allergen stimulation (Figure 3C), whereas AuNPs 0.4 μg/mL (Figure 3D) and 4 μg/mL (Figure 3E) significantly inhibited allergen-induced mucus secretion. Quantitative morphometric analyses confirmed that treatment with Budesonide did not alter mucus exacerbation in this model, but AuNPs have inhibited this output (Figure 3K). Figure 3 also shows that lung sections stained with the Gömöri trichrome revealed that an increased deposit of extracellular matrix occurred in the peribronchiolar region of mice stimulated with the allergen (Figure 3G) compared with the negative controls (Figure 3F). AuNPs 0.4 μg/mL (Figure 3I) and 4 μg/mL (Figure 3J) significantly reduced the fibrotic response, whereas Budesonide (Figure 3G) was inactive. Quantitative data on mucus production and airway remodeling are shown in Figure 3K and Figure 3L, respectively.

### 3.4. Effect of AuNPs but Not Budesonide on Cytokine and Chemokine Generation Caused by Allergen Provocation

We then examined the effect of nebulized Budesonide or AuNPs on the lung generation of cytokines and chemokines in the long-term stimulation by allergen of A/J mice. We found that the lung tissue levels of IL-4 (Figure 4A), IL-13 (Figure 4B), IL-17 (Figure 4C), CCL11/eotaxin-1 (Figure 4D), CCL24/eotaxin-2 (Figure 4E), CXCL1/KC (Figure 4F) and CCL17/TARC (Figure 4G) increased in allergen-stimulated mice as compared with the sham-stimulated ones. Accordingly, AuNPs but not Budesonide were active in this long-term murine model of asthma.

### 3.5. Effect of AuNPs on Redox Imbalance Related to Corticosteroid Resistance in A/J Mice

**Figure 5:**
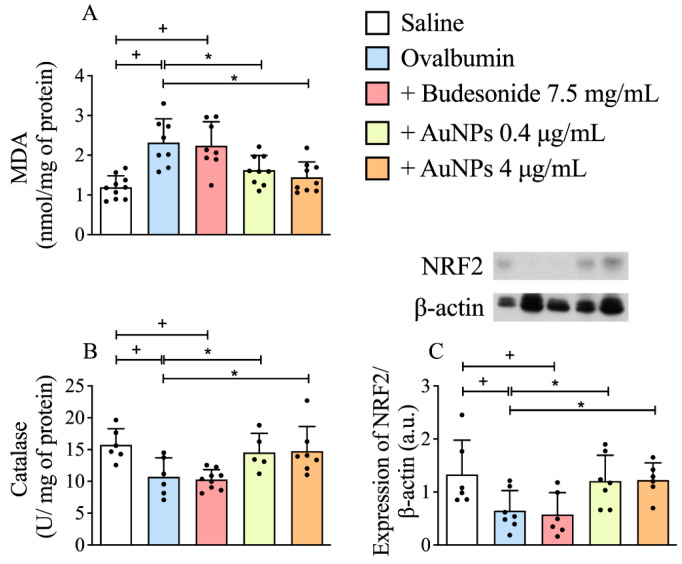
The effect of nebulized Budesonide or gold nanoparticles (AuNPs) on changes in oxidative stress markers following ovalbumin intranasal instillation given once a week for 9 weeks. Biochemical analyses of malondialdehyde (MDA) (**A**) and antioxidant enzyme catalase (**B**), as well as Western blotting evaluation on nuclear factor erythroid 2-related factor 2 (NRF2) expression (**C**), were assessed in whole-lung homogenates. Data are expressed as mean ± SD ((Each dot represents an individual mouse; *n* = 6–7 for all groups). + *p* ˂ 0.05 as compared with the saline group, * *p* ˂ 0.05 as compared with the ovalbumin-challenged group.

We found that intranasal allergen stimulation led to a reduction in catalase activity (Figure 5B) accompanied by a rise in the oxidative damage in lipids, measured by MDA formation (Figure 5A) in lung tissue of allergen-provoked mice as compared to those obtained from sham-stimulated ones. We also showed that allergen provocation reduced the levels of a master regulator of the antioxidant response NRF2 (Figure 5C), as attested by the Western blotting assay. Concerning the redox parameters monitored in this study, nebulization of sensitized mice with AuNPs (0.4 μg/mL and 4 μg/mL) but not Budesonide (7.5 mg/mL) significantly inhibited all the changes elicited by prolonged allergen exposure (Figure 5A–C).

### 3.6. Effect of AuNPs on the Activated PI3Kδ/HDAC2 Pathway following Long-Term Allergen Exposure in A/J Mice

**Figure 6:**
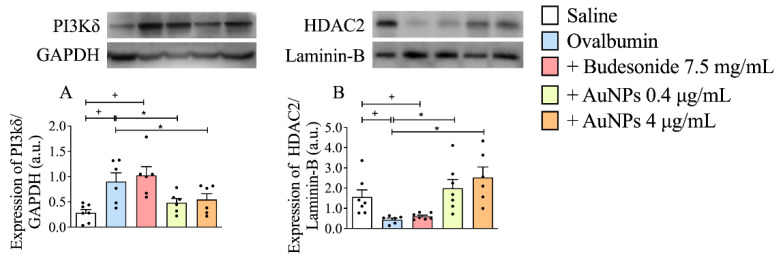
The effect of nebulized Budesonide or gold nanoparticles (AuNPs) on changes in phosphoinositide 3-kinases δ (PI3Kδ) and histone deacetylase 2 (HDAC2) expression in lung tissue samples. The whole-lung extract was analyzed by Western blotting for PI3Kδ (**A**), while HDAC2 expression (**B**) was assessed in the nuclear extract of the lung tissue. The intensity of either PI3Kδ and HDAC2 expression was quantified by densitometry and presented as relative intensity of glyceraldehyde-3-phosphate dehydrogenase (GAPDH) and Lamin-B, respectively. Data are expressed as mean ± SD ((Each dot represents an individual mouse; *n* = 6 − 7 for all groups). + *p* ˂ 0.05 as compared with the saline group, * *p* ˂ 0.05 as compared with the ovalbumin-challenged group.

Several mechanisms were linked to the development of corticosteroid resistance, including the excessive activation of PI3Kδ and the reduction of HDAC2 activity [15,21]. We showed that the exposure of sensitized mice to allergen led to an increase in the PI3Kδ lung tissue expression (Figure 6A) in close relationship with a decrease in the HDAC2 nuclear levels (Figure 6B) as compared to sham-challenged mice. The treatment with AuNPs reduced the PI3Kδ levels and restored the HDAC2 levels, whereas Budesonide was ineffective (Figure 6A,B).

## 4. Discussion

Corticosteroid resistance confers a much higher risk of life-threatening and poor outcomes for asthma patients and is a significant barrier to therapeutically managing the disease [17,23,24]. Murine models of allergic asthma offer opportunities to dissect inflammatory pathways and develop new therapeutics, but they rarely replicate pathological features of chronic inflammation or steroid-insensitive asthma [25,26]. The results obtained in the current study indicate that delivering allergen by instillation into the nose in A/J mice for 9 weeks helps evaluate features of persistent asthma-like changes and steroid resistance. We found that persistent pulmonary allergic dysfunction was refractory to the treatment with Budesonide but responded to nebulized AuNPs in this model. AuNPs treatment also restored HDAC2 nuclear levels and promoted a balanced redox environment by preserving the NRF2 pathway. These findings suggest that AuNPs hold promising perspectives as an alternative in drug development for difficult-to-treat asthma.

Animal modeling systems that accurately resemble disease pathophysiology are pivotal to developing new therapies. Remarkably, Shinagawa and Kojima reported that when mice of the strains BALB/c, C57BL/6, C3H/HeJ, and A/J were subjected to a long-term protocol of allergen stimulations, persistent lung eosinophilic inflammation and airway remodeling were observed only in A/J mice [26]. We used a similar protocol to confirm that A/J mice responded with continuous pathological features, such as lung inflammation, airway hyper-reactivity, mucus hypersecretion, and peribronchiolar fibrosis. It is noteworthy that T cell tolerance occurred in BALB/c but not A/J mice when both were chronically stimulated [27]. Inhalation of ovalbumin leads to tolerance mediated by Foxp3+ CD4+ T regulatory cells (Tregs) in mice [28]. The immunosuppressive role of these cells is attested by the transfer of Tregs that can suppress the main features of experimentally induced asthma via secretion of IL-10 and TGF-β [29]. Remarkably, despite showing a comparable strong lung inflammatory response and airway hyperresponsiveness to ovalbumin provocation, Foxp3+ Treg cells obtained from lung draining lymph nodes expanded significantly lesser following allergen stimulation when obtained from A/J as compared to those from BALB/c mice [30]. These findings reinforce the interpretation that the incapacity to develop T cell tolerance may be pivotal for the persistent inflammatory response exhibited by A/J mice.

Asthma is a heterogeneous disease that can be subdivided into categories (phenotypes) based on characteristics, such as the predominance of eosinophils or neutrophils in the lung inflamed tissue [24,31]. We observed that allergen-induced leukocyte infiltration into the lung of A/J mice was accounted for by elevations in the numbers of mononuclear cells (7-fold), eosinophils (214-fold), and neutrophils (356-fold) compared to baseline levels (sham-challenged mice), pointing out a slight predominance of neutrophils over eosinophils in the leukocyte infiltrate into the damaged lung in this model. The dual Th2- Th17-type profile of such a response appeared reflected in the cytokines and chemokines generated within the lungs. The generation of pro-neutrophilic factors such as IL-17 and CXCL1/KC increased, and elevated levels of pro-eosinophilic factors such as IL-4, IL-13, CCL11/eotaxin-1 and CCL24/eotaxin-2 were also apparent.

Prior works involving animal models and clinical studies have emphasized the role of eosinophils and neutrophils as effector cells in the pathogenesis of severe asthma [25,32,33]. Our findings align with many others where a close correlation between the location and activation of these cells with pathological changes such as airway hyperreactivity, mucus secretion, and peribronchiolar fibrosis were observed [25,32,33]. Steroid anti-inflammatory drugs are often effective inhibitors of airway eosinophilia, but higher doses are needed to block this event under severe asthma [34]. Uncontrolled asthmatics may also exhibit persistent airway eosinophilia even under prolonged systemic steroid therapy [35]. Airway neutrophilia has also been associated with asthma exacerbations and steroid insensitivity [36,37]. Accordingly, an early study documented higher neutrophil than eosinophil numbers in the lung tissue of patients who had died from an asthma exacerbation [38].

It is worth noting that the dual eosinophilic and neutrophilic inflammatory response and all the other inflammatory structural and functional changes triggered by long-lasting allergen stimulation remained unchanged in A/J mice subjected to long-term treatment with nebulized Budesonide, suggesting that this is an appropriate long-term model of asthma with steroid resistance. Evidence that A/J mice tend to express corticosteroid insensitivity following repeated inflammatory stimulations has also been observed in short-term models of non-allergic [39,40] and allergic [22] lung inflammatory conditions. Strikingly, the treatment with nebulized Budesonide was fully active against allergen-induced airway hyperresponsiveness as a short-term protocol of allergen provocation (four instead of nine weeks) was employed, indicating that A/J mice are not naturally steroid-insensitive. They become insensitive as the disease’s manifestations persist under prolonged challenges [22,39,40].

Because intranasal administration of AuNPs prevents pivotal pathological changes in short-term models of steroid-sensitive asthma [15], the current study examined whether the pathological features of Budesonide-resistant asthma can be affected by nebulized AuNPs. Many studies show that AuNPs possess significant anti-inflammatory and anti-oxidative properties [41,42]. The interventional treatment with AuNPs effectively ameliorates all functional and structural changes monitored in the long-term model of asthma in A/J mice, including airway hyperreactivity, mucus hypersecretion, and peribronchiolar fibrosis. The protective effect of AuNPs correlated with significant inhibition of pro-inflammatory cytokines and chemokines, such as IL-4, IL-13, IL-17, CXCL1/KC, CCL11/eotaxin-1, and CCL24/eotaxin-2 quantified in the lung tissue. Nevertheless, no statistical differences were detected for the two doses of AuNPs, except for CCL24/eotaxin-2 which was sensitive only to the higher one. It also correlated with the blockade of the lung tissue inflammatory infiltrate of neutrophils, eosinophils, and mononuclear cells, reducing those cell levels by about 90, 93, and 77%, respectively, at the higher dosage.

Both inflammation and oxidative stress are critical players in the pathophysiology of steroid-insensitive asthma [33,43]. In line with this concept, we found that intranasal allergen stimulation led to a marked rise in oxidative damage, measured by MDA formation in the lung tissue. Increased formation of reactive oxygen and nitrogen species is commonly linked with the loss of antioxidant capacity in patients with severe asthma and COPD [33]. Indeed, we showed that allergen provocation caused a reduction in catalase activity. It also reduced the lung tissue expression of the cytoprotective transcription factor NRF2. Inflammation and oxidant/antioxidant imbalances are causally linked to severe asthma pathological features, such as airway hyperresponsiveness, mucus exacerbation, and adverse lung remodeling [33,44,45]. When we assessed the effectiveness of either AuNPs or Budesonide nebulization on the allergen-induced oxidative stress response, only the former treatment was effective. AuNPs treatment rescued the reduced catalase activity and decreased MDA levels in the lung tissue, which are biomarkers of oxidative tissue damage, respectively. We observed that nebulized AuNPs also recovered the steady-state lung tissue levels of NRF2.

NRF2 is a redox-sensitive transcription factor able to transform oxidative stress signals into genes and proteins implicated in cellular antioxidant defense and anti-inflammatory effects [46]. It is noteworthy that allergen-induced asthma features, including airway eosinophilia, mucus hypersecretion, and airway hyper-reactivity are exacerbated in NRF2-null mice, with reduction in the expression of anti-oxidant genes, and elevation of type 2 cytokines [47]. Strikingly, patients with severe asthma are marked by low levels of both NRF2 and HDAC2 mRNA levels in peripheral blood mononuclear cells, reinforcing the interpretation that the decrease in the expression of these genes is a pivotal determinant of susceptibility to asthma exacerbations [48].

Prior research demonstrated that activation of the NRF2 pathway is part of the mode of action of gold drugs for treating rheumatoid arthritis [13]. The activation of NRF2 by 5 nm AuNPs was reported in CaCo-2 cells [49] and human vascular endothelial cells [50]. Strikingly, a short incubation time (0.5 h) of AuNPs was enough to evoke NRF2 nuclear translocation in keratinocytes [51]. Therefore, the possibility does exist that the interconnected anti-inflammatory and antioxidative effect of AuNPs, noted in the current model of steroid-resistant asthma, is accounted for by the up-regulation of the NRF2 pathway. Still, additional experiments are required to clarify the mechanism implicated in this effect.

Finally, one key mechanism of steroid resistance in COPD and severe asthma patients is reducing histone deacetylase-2 (HDAC2) levels. Oxidative stress activates PI3Kδ, which phosphorylates downstream kinases, such as Akt, resulting in the phosphorylation and ubiquitination of HDAC2, a pivotal enzyme in the inflammatory gene transcription blockade by glucocorticoids [17,33]. Like NRF2, HDAC2 is expressed in airway epithelium and inflammatory cells and can inhibit allergen-induced airway inflammation by suppressing IL-17A production in mice [52]. Our findings revealed that AuNPs, but not Budesonide, inhibited the PI3Kδ expression in lung tissue, contributing to the observed HDAC2 preservation in this model. The rescue of the nuclear content of a negative regulator of histones acetylation HDAC2, which appeared reduced in untreated allergen-challenged mice, suggests that AuNPs have the potential to unlock the corticosteroid resistance. New experiments are underway to confirm this hypothesis.

**Figure A1:**
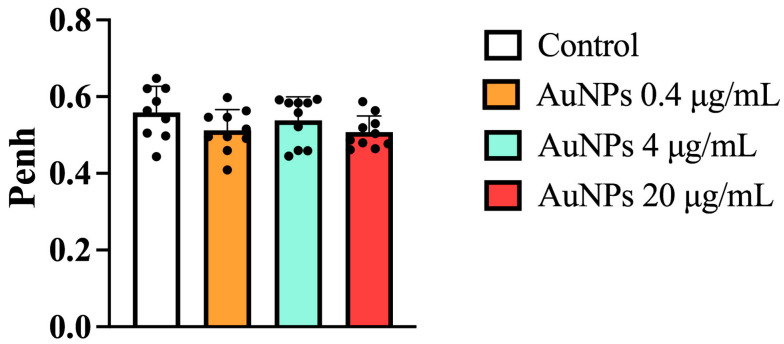
Effect of treatment with nebulized AuNPs on healthy A/J mice. Naïve A/J mice were subjected to AuNP nebulization (0.4; 4, and 20 µg/mL) 3 times a week for 7 consecutive weeks. Airway resistance was measured using non-invasive plethysmography and expressed as Pause Enhancement (Penh). Data are expressed as mean ± SD of 10 mice for all groups except the saline group (*n* = 9). Each dot represents an individual mouse.

The aforementioned pharmacological properties of the 13 nm citrate conjugate AuNP add support to the therapeutical use of gold nanoparticles in severe asthma, but to achieve this in a safe and effective manner, some putative limitations must be carefully considered. AuNPs are prone to aggregation and protein opsonization *in vivo*, which may accelerate clearance by phagocytosis or filtration in organs such as the liver and kidney, resulting in decreased tissue retention time and bioavailability [53]. AuNP toxicity in biological systems is another debated issue [48]. Using concentrations of nebulized AuNPs up to 15-fold higher than the highest dosage employed in the current study, we failed to detect significant changes in the lung airway resistance as attested by non-invasive barometric plethysmography in naïve A/J mice (Appendix A, Figure A1). In addition, no lung histopathological changes were noted on days 1, 3, and 28 after exposing rats to inhaled AuNPs (13 nm) for five days [54]. In contrast, the in vivo assessment of a wide size range of citrate-capped AuNPs in mice (8 mg/kg/week, i.p.) revealed that the smallest sizes (3 and 5 nm) and the largest (50 and 100 nm) were not toxic, but the intermediate size range (8-37 nm) caused significant sickness, including loss of appetite, weight loss, and a shorter average lifespan [55]. While these three studies had several differences concerning experimental protocols and animal employed, more data on AuNP toxicity are required.

## 5. Conclusions

**Figure 7:**
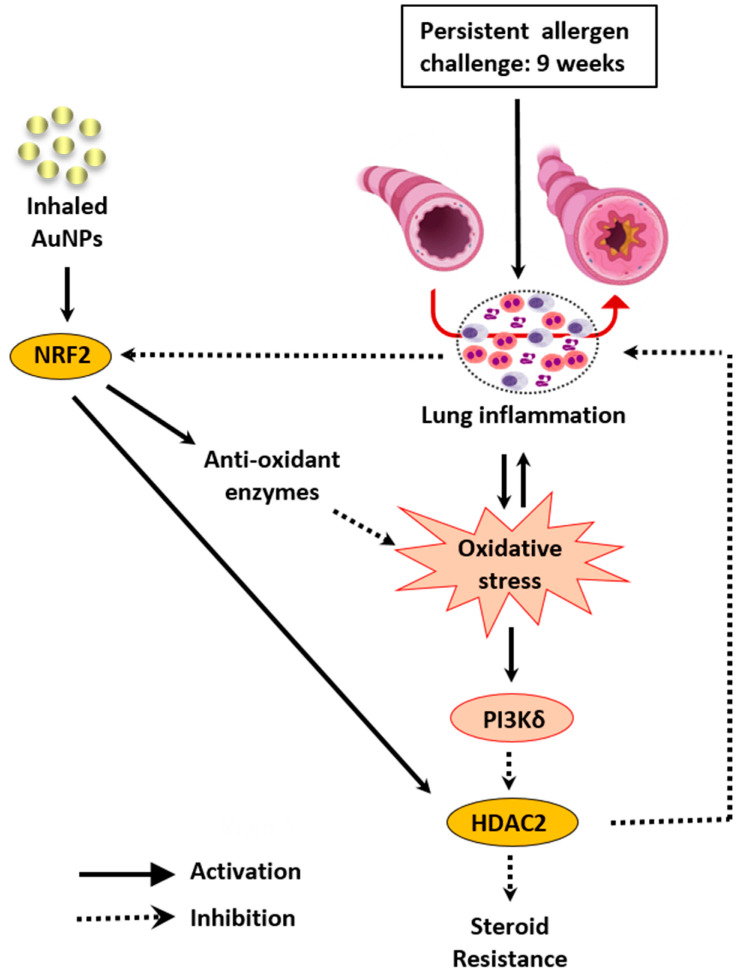
Schematic illustration of how gold nanoparticles (AuNPs) might be working in this murine model of glucocorticoid-insensitive asthma. Persistent allergen provocation of A/J mice causes chronic inflammation characterized by neutrophil and eosinophil infiltration, airway hyper-reactivity, mucus exacerbation, and lung remodeling. Oxidative stress via phosphoinositide 3-kinases δ (PI3Kδ) decreases GC sensitivity by down-regulating histone deacetylase 2 (HDAC2), a co-factor of glucocorticoid suppressive genomic action, which has a pivotal role in inhibiting allergen-induced lung inflammation. AuNPs can activate nuclear factor erythroid 2-related factor 2 (NRF2), whose expression appeared reduced in untreated and Budesonide-treated asthmatic mice but not in those asthmatic mice subjected to AuNP inhalation. Activation of NRF2 can up-regulate antioxidant enzymes and rescue HDAC2 baseline levels. Inhaled AuNPs decreased several steroid-insensitive asthma changes, including lung inflammation and adverse remodeling.

This study investigated whether inhaled naked 13 nm AuNPs might interfere with allergen-induced asthma changes in a murine model of steroid-resistant asthma, and if they did work, what would be their putative mode of action. As depicted in Figure 7, these findings suggest that ovalbumin-sensitized A/J mice subjected to a long-term protocol of repeated intranasal ovalbumin provocations evolve to a chronic asthma-like condition that is unresponsive to Budesonide treatment. All pathological changes reproduced in this model are sensitive to AuNPs given by nebulization, in a context associated with down-regulation of oxidative stress and rescue of HDAC2 levels. The data also suggest that stimulation of antioxidative response through activation of NRF2 may be a pharmacologically important part of the actions of AuNPs in this model, providing novel perspectives for treating steroid-insensitive asthma and COPD at the interface of biological systems and nanomedicine.

## Figures and Tables

**Figure A2:**
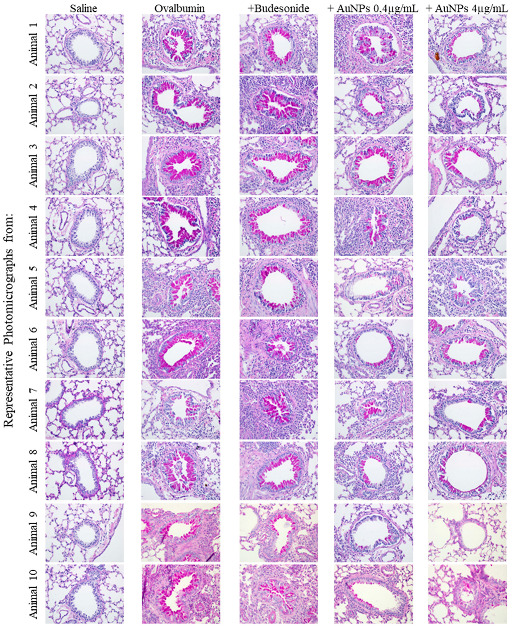
Effect of nebulized budesonide or AuNPs on mucus production and airway remodeling following ovalbumin intranasal instillation given once a week for 9 weeks. Photomicrographs were taken of representative airways (animals 1 to 10) stained by periodic acid-Schiff (Mucus analysis) or Gömöri trichrome (Extra cellular matrix deposit) from ovalbumin-sensitized mice challenged with saline (Saline), challenged with ovalbumin (Ovalbumin), challenged with ovalbumin and treated with budesonide (+Budesonide), challenged with ovalbumin and treated with AuNPs (+0.4 μg/mL AuNP) and challenged with ovalbumin and treated with AuNPs (+4 μg/mL AuNPs).

**Figure A3:**
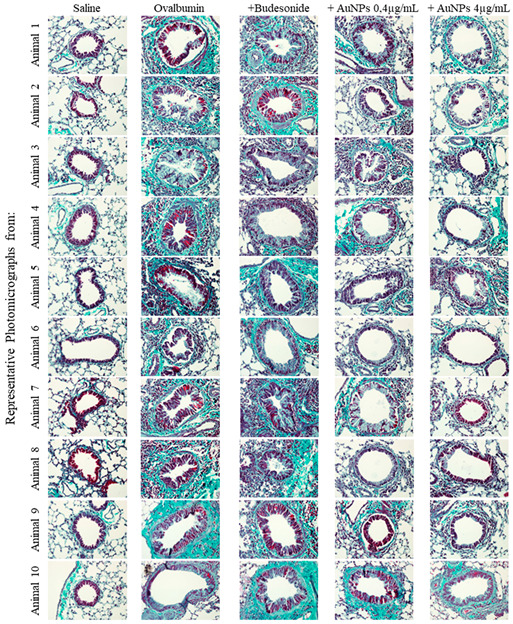
Effect of nebulized budesonide or AuNPs on mucus production and airway remodeling following ovalbumin intranasal instillation given once a week for 9 weeks. Photomicrographs were taken of representative airways (animals 1 to 10) stained by Gömöri trichrome (Extra cellular matrix deposit) from ovalbumin-sensitized mice challenged with saline (Saline), challenged with ovalbumin (Ovalbumin), challenged with ovalbumin and treated with budesonide (+Budesonide), challenged with ovalbumin and treated with AuNPs (+0.4 μg/mL AuNP) and challenged with ovalbumin and treated with AuNPs (+4 μg/mL AuNPs).

**Figure A4:**
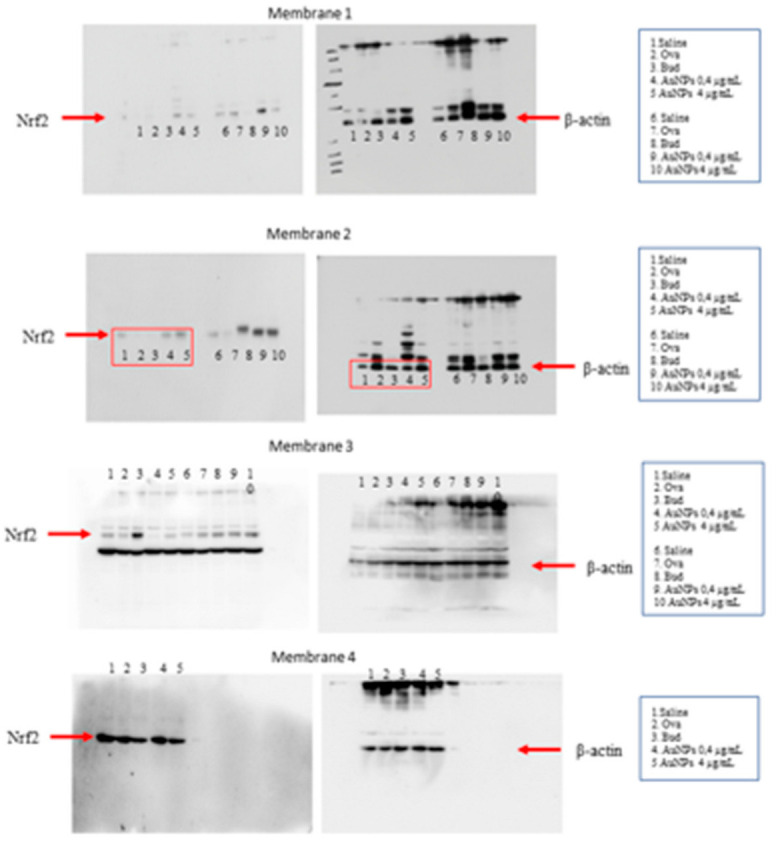
Upload of full gels for all Western blot images used to quantify Nrf2 as shown in Figure 5C. Red rectangles indicate lanes presented as representative blots in Figure 5C.

**Figure A5:**
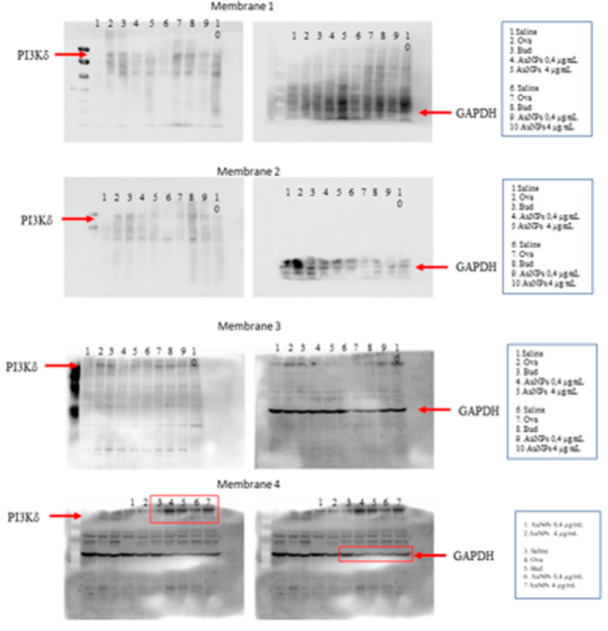
Upload of full gels for all Western blot images used to quantify PI3Kδ as shown in Figure 6A. Red rectangles indicate lanes presented as representative blots in Figure 6A.

**Figure A6:**
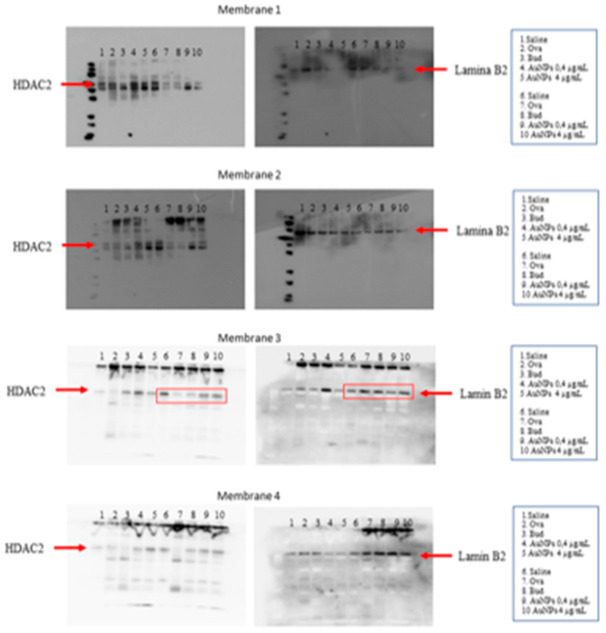
Upload of full gels for all Western blot images used to quantify HDAC2 as shown in Figure 6B. Red rectangles indicate lanes presented as representative blots in Figure 6B.

## Data Availability

All the data supporting current reported results will be available on demand.

## Supplementary Materials

The following supporting information can be downloaded at: https://www.mdpi.com/article/10.3390/antiox11091659/s1, Figure S1: Effect of treatment with nebulized Budesonide on airway hyper-reactivity following the protocol of ovalbumin intranasal instillation given once a week for 4 weeks; Supplementary data.
