# Supplementary material for: Gold Nanoparticles Inhibit Steroid-Insensitive Asthma in Mice Preserving Histone Deacetylase 2 and NRF2 Pathways
Source: Antioxidants (Basel). 2022 Aug 26;11(9):1659. doi: 10.3390/antiox11091659 (PMC9495660; doi:10.3390/antiox11091659)
Supplement: Supplementary file 1 [file antioxidants-11-01659-s001.zip › Supplementary data.pdf]

Antioxidants – Supplementary Material

Gold Nanoparticles Inhibit Steroid-Insensitive Asthma in Mice Preserving Histone Deacetylase 2 and NRF2 Pathways

Magda F. Serra [1], Amanda C. Cotias [1], Andreza S. Pimentel [1], Ana Carolina S. de Arantes [1], Ana Lucia A. Pires [1], Manuella Lanzetti [2], Jandir M. Hickmann [3], Emiliano Barreto [4], Vinicius F. Carvalho [1], Patrícia M. R. e Silva [1], Renato S. B. Cordeiro [1] and Marco Aurélio Martins [1,*]

[1] Laboratory of Inflammation, Oswaldo Cruz Institute, Oswaldo Cruz Foundation, Rio de Janeiro 21040-360, RJ, Brazil; magda.fraguas@prof.abeugraduacao.com.br (M.F.S.); accotias@ioc.fiocruz.br (A.C.C.); andreza.spimentel@gmail.com (A.S.P.); aarantes@ioc.fiocruz.br (A.C.S.d.A.); anapires@ioc.fiocruz.br (A.L.A.P.); vfrias@ioc.fiocruz.br (V.F.C.); patmar@ioc.fiocruz.br (P.M.R.e.S.); cordeiro@ioc.fiocruz.br (R.S.B.C.)

[2] Institute of Biomedical Science, Federal University of Rio de Janeiro, Rio de Janeiro 21941-902, RJ, Brazil; manuellalanzetti@icb.ufrj.br

[3] Institute of Physics, Federal University of Rio Grande do Sul, Porto Alegre 91509-900, RS, Brazil; jmh@optma.org

[4] Laboratory of Cell Biology, Federal University of Alagoas, Maceió 50072-900, AL, Brazil; emilianobarreto@icbs.ufal.br

* Correspondence: mmartins@ioc.fiocruz.br; Tel.: +55-21-2562-1358

Figure S1

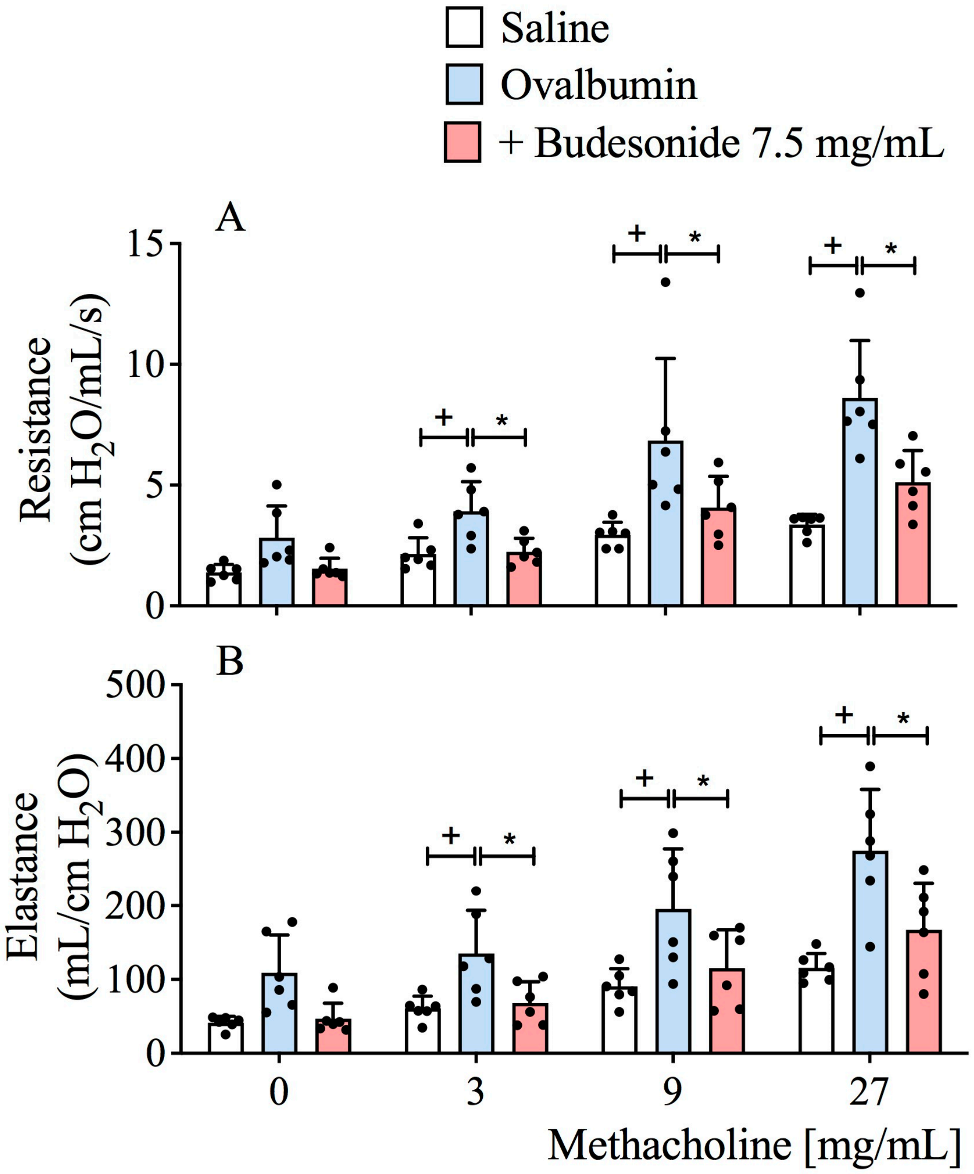

Figure S1 - Effect of treatment with nebulized Budesonide on airway hyper-reactivity following the protocol of ovalbumin intranasal instillation given once a week for 4 weeks. The treatment was given 1 h before allergen provocation at weeks 3 and 4. Airway responsiveness was measured by changes in lung resistance and elastance induced by aerosolization of increasing concentrations of methacholine 24 h after the last antigen challenge. Data are expressed as mean ± SD (Each dot represents an individual mouse; n is at least 6 for all groups). $^{+}P<0.05$ as compared with the Saline group, $*P<0.05$ as compared with the ovalbumin-challenged group.
