# Supplementary figures and images for: Gold Nanoparticles Inhibit Steroid-Insensitive Asthma in Mice Preserving Histone Deacetylase 2 and NRF2 Pathways
Source: Antioxidants (Basel). 2022 Aug 26;11(9):1659. doi: 10.3390/antiox11091659 (PMC9495660; doi:10.3390/antiox11091659)

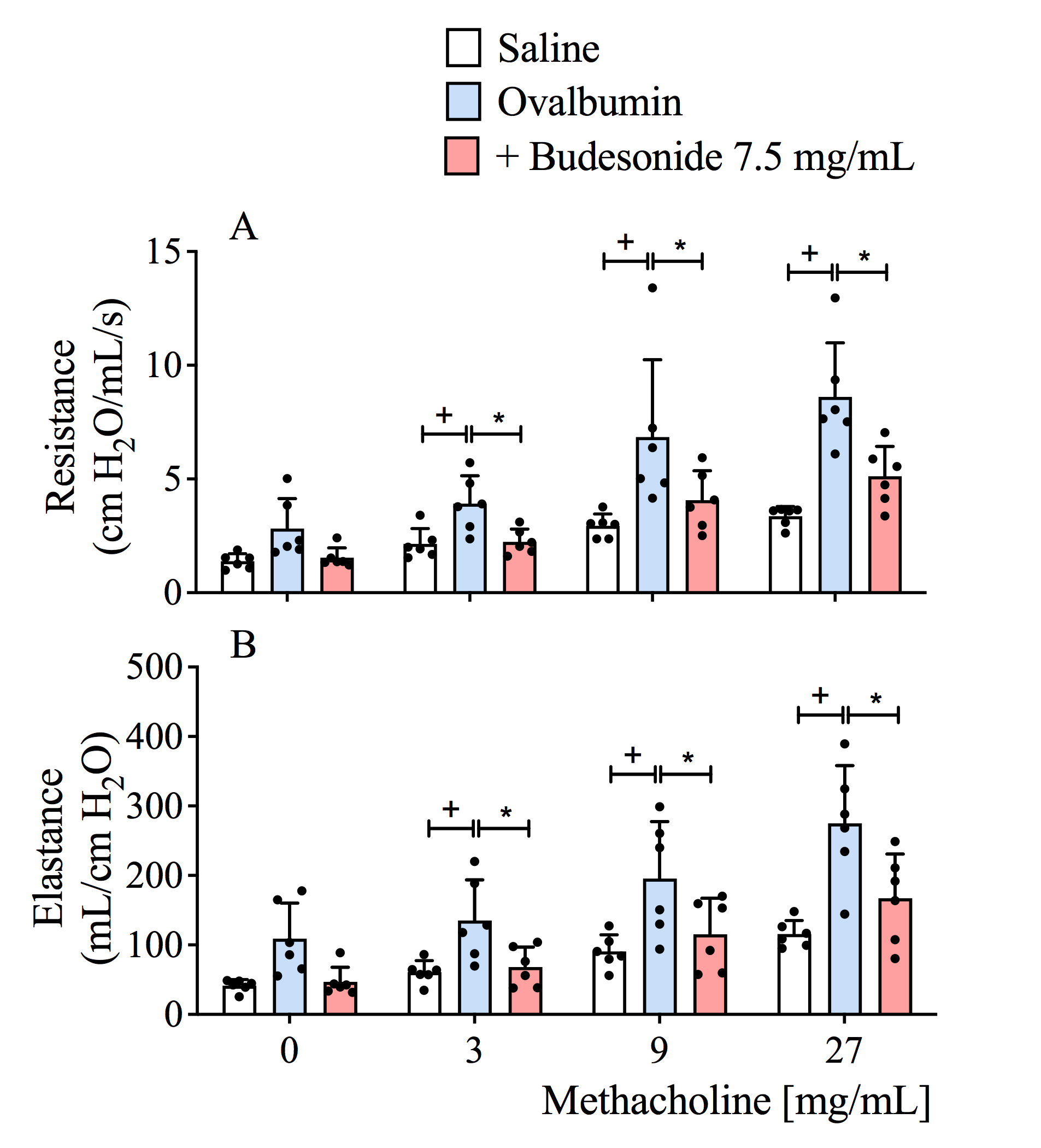

Supplement: Supplementary file 1 [file antioxidants-11-01659-s001.zip › Figure S1.tiff]
